# Comparison of logistic-regression based methods for simple mediation analysis with a dichotomous outcome variable

**DOI:** 10.1186/s12874-018-0654-z

**Published:** 2019-01-21

**Authors:** Judith J. M. Rijnhart, Jos W. R. Twisk, Iris Eekhout, Martijn W. Heymans

**Affiliations:** 1 Department of Epidemiology and Biostatistics, Amsterdam UMC, Location VU University Medical Center, P.O. Box 7057, 1007 MB Amsterdam, The Netherlands; 2 Department of Child Health, Netherlands Organization for Applied Scientific Research TNO, Schipholweg 77, 2316 ZL Leiden, The Netherlands

**Keywords:** Mediation analysis, Indirect effect, Proportion mediated, Multiple regression, Structural equation modeling, Potential outcomes framework, Dichotomous outcome

## Abstract

**Background:**

Logistic regression is often used for mediation analysis with a dichotomous outcome. However, previous studies showed that the indirect effect and proportion mediated are often affected by a change of scales in logistic regression models. To circumvent this, standardization has been proposed. The aim of this study was to show the relative performance of the unstandardized and standardized estimates of the indirect effect and proportion mediated based on multiple regression, structural equation modeling, and the potential outcomes framework for mediation models with a dichotomous outcome.

**Methods:**

We compared the performance of the effect estimates yielded by the three methods using a simulation study and two real-life data examples from an observational cohort study (*n* = 360).

**Results:**

Lowest bias and highest efficiency were observed for the estimates from the potential outcomes framework and for the crude indirect effect *ab* and the proportion mediated *ab*/(*ab* + *c’*) based on multiple regression and SEM.

**Conclusions:**

We advise the use of either the potential outcomes framework estimates or the *ab* estimate of the indirect effect and the *ab*/(*ab* + *c’*) estimate of the proportion mediated based on multiple regression and SEM when mediation analysis is based on logistic regression. Standardization of the coefficients prior to estimating the indirect effect and the proportion mediated may not increase the performance of these estimates.

## Background

**Fig. 1:**
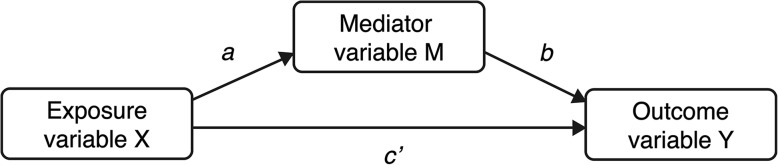
Path diagram of a relatively simple mediation model

Epidemiologists are often interested in the relationship between an exposure and an outcome. The pathways underlying such a relationship, however, often remain unknown. These unknown pathways can be assessed using mediation analysis. Mediation analysis decomposes the total effect of an exposure on an outcome (*c* path) into a direct effect (*c’* path in Fig. 1) and indirect effect (*a* and *b* paths in Fig. 1). This makes mediation analysis especially useful for disentangling mechanisms of disease development, and for identifying important intermediate factors in establishing treatment effects [1].

In simple mediation models, as visualized in Fig. 1, the indirect effect can be calculated as either the product of the *a* and *b* paths (i.e. the product-of-coefficients approach), or as the difference between the *c* and *c’* paths (i.e. the difference-between-coefficients approach). In addition, a proportion mediated can be calculated using one of the following approaches: 1) divide the indirect effect *ab* by the sum of *ab* and the direct effect *c’*, 2) divide the indirect effect *ab* by the total effect *c*, or 3) subtract the direct effect *c’* divided by the total effect *c* from 1 [2]. Multiple regression analysis and Structural Equation Modeling (SEM) can both be used to estimate the paths in Fig. 1.

In general, when the mediator and outcome are both continuous, the product-of-coefficients and difference-between-coefficients approach for calculating the indirect effect and the three approaches for calculating the proportion mediated will lead to the same results [2]. However, previous simulation studies showed that the different estimates of the indirect effect and proportion mediated will no longer coincide when the outcome is dichotomous and logistic regression analysis is used to estimate the paths in Fig. 1 [2, 3].

To limit the discrepancies between the different approaches for calculating the indirect effect and proportion mediated, several authors proposed to standardize the logistic regression coefficients. MacKinnon and Dwyer [3] proposed the use of *y*-standardization, Kenny [4] proposed the use of full-standardization, and MacKinnon and colleagues [2] proposed the use of the standardized logistic solution. Standardization equalizes the scales of the coefficients across multiple different logistic regression models to make the coefficients comparable. Another regression-based method that has been proposed for estimating the indirect effect and proportion mediated is the potential outcomes framework. This framework provides definitions of causal effects, which can be used to decompose the total effect into a causal direct and indirect effect without requiring standardization of the coefficients [5].

It remains unclear which (standardized) approach for calculating the indirect effect and proportion mediated is preferred in what situation, and when the potential outcomes framework should be preferred over multiple regression and SEM. Therefore, our aim is to show the relative performance of the unstandardized and standardized estimates of the indirect effect and proportion mediated based on multiple regression, SEM, and the potential outcomes framework for models with a dichotomous outcome and 1) a continuous mediator, and 2) a dichotomous mediator.

## Methods

### Aim

The aim of this paper is to show the relative performance of the unstandardized and standardized estimates of the indirect effect and proportion mediated based on multiple regression, SEM, and the potential outcomes framework for models with a dichotomous outcome and 1) a continuous mediator, and 2) a dichotomous mediator.

### Simulation set up

To assess the relative performance of the compared methods, we simulated data for two types of mediation models with a dichotomous outcome; 1) with a continuous normally distributed mediator with a mean of 0 and variance of 1, and 2) with a dichotomous mediator. For both the dichotomous mediator and the dichotomous outcome three prevalence rates were simulated: 0.10, 0.30, and 0.50. Therefore, three conditions were created with a continuous mediator and dichotomous outcome, and nine conditions with a dichotomous mediator and dichotomous outcome. The exposure was a normally distributed continuous variable with a mean of 0 and a variance of 1 in all conditions. The dichotomous mediator and outcome where generated directly from a logistic model. Furthermore, in each condition the *a*, *b*, and *c’* paths in the underlying population model were set to 0.6, reflecting a medium-to-large effect size [2]. The standardized effect estimates were yielded by standardizing the crude effect estimates in each simulated sample.

Table 1 provides an overview of the true underlying estimates of the indirect effect for each simulated condition. The true values for the standardized effect estimates were calculated by applying the standardization equations to the true underlying crude effect estimates [2, 6]. In all conditions the true proportion mediated in multiple regression and SEM equaled 0.375. For the potential outcomes framework the true proportion mediated was 0.375 for the condition with a continuous mediator, and 0.119, 0.127, and 0.073 for the conditions with a dichotomous mediator with a prevalence of 0.5, 0.3, and 0.1 respectively. For each condition, 500 simulated samples of 1000 subjects were generated. All simulations were performed using STATA statistical software release 14 [7].

**Table 1:** True underlying indirect effect estimates for each simulated condition

	Continuous mediator	Dichotomous mediator prevalence		
		0.1	0.3	0.5
Multiple regression/SEM				
crude	0.360	0.360	0.360	0.360
*y*-standardization	0.168	0.098	0.097	0.096
Full-standardization	0.196	0.048	0.044	0.048
Standardized logistic solution	0.360	NA	NA	NA
Potential outcomes framework	0.360	0.047	0.087	0.081

*Abbreviations: NA* not available

#### Performance measures

The performance of each method was evaluated using the (absolute) bias and Mean Squared Error (MSE). The bias is calculated as the average difference between the effect estimates in the simulated samples and the true underlying effect. A negative bias indicates that the method underestimates the true underlying effect, and a positive bias indicates that the method overestimates the true underlying effect. The MSE is calculated as the average squared difference between the effect estimates in the simulated samples and the true underlying effect. The MSE represents the amount of variasbility in the effect estimates. So the higher the MSE, the higher the variability is and thus the lower the efficiency of the method is [8].

### Real-life data examples

To demonstrate the similarities and differences between the effect estimates yielded by the compared methods, two real-life data examples from a longitudinal observational cohort study were used. The aim of this longitudinal study was to follow up the natural growth, health, and lifestyle in a representative sample of 698 Dutch adolescents [9]. In total, ten measurement rounds were performed between 1976 and 2006. Our data example was based on the measurement round in the year 2000, when the participants were in their 30s. The exposure was the sum of four skinfolds in centimeters, which is an indicator of body fatness. The outcome was carotid distensibility (CD), which is a measure of carotid artery elasticity. The association between the sum of four skinfolds and CD was thought to be mediated by heart rate. Heart rate was analyzed as both a continuous and a dichotomous measure. Heart rate and CD were dichotomized by splitting them at the median. The analytical cohort consisted of 360 participants. The statistical analyses were performed with STATA statistical software release 14 [7]. The STATA package ‘paramed’ was used to apply the potential outcomes framework [10].

### Methods for statistical mediation analysis

#### Multiple regression and SEM

Equations 1, 2, and 3 can be used to fit simple mediation models, as shown in Fig. 1, with multiple regression and SEM [11]. The difference between multiple regression and SEM is that with multiple regression separate models are fitted for each equation, whereas with SEM eqs. 2 and 3 can be fitted simultaneously in one model [12]. When the mediator is continuous, eqs. 1 and 3 are fitted with logistic regression and eq. 2 with linear regression. When the mediator is dichotomous, all equations are fitted with logistic regression.

1 $$ \mathrm{Y}={i}_1+c\mathrm{X} $$

2 $$ \mathrm{M}={i}_2+a\mathrm{X} $$

3 $$ \mathrm{Y}={i}_3+{c}^{\prime}\mathrm{X}+b\mathrm{M} $$

Where, in eq. 1, Y represents the outcome, and *c*X represents the slope of the exposure. In eq. 2, M represents the mediator, and *a*X represents the slope of the exposure. In eq. 3, Y represents the outcome, *c*^′^X represents the slope of the exposure, and *b*M represents the slope of the mediator. In all equations *i* represents the intercepts.

The discrepancies between the different estimates of the indirect effect and proportion mediated in multiple regression and SEM are caused by a change of scales of the coefficients in nested logistic models [13]. This change of scales happens when variables are added to a logistic regression model, and even happens when these variable are not related to the independent variable in the model. Because of this change of scales of the coefficients in logistic regression analysis after adding a potential mediator that is highly related to the outcome, the indirect effect and proportion mediated based on the crude coefficients from logistic regression might not be reliable indicators for the presence of a mediated effect. Even when there truly is mediation, the magnitude of the estimates of the indirect effect and proportion mediated will be affected by the change of scales of the coefficients.

To equalize the scales of the coefficients across logistic regression models, *y*-standardization, full-standardization, and the standardized logistic solution have been proposed [2–4]. Both *y*-standardization and full-standardization can be applied regardless of whether the mediator is continuous or dichotomous, however with a continuous mediator the *a* coefficient does not have to be standardized [6, 14]. The standardized logistic solution can only be applied when the mediator is continuous [2]. The three standardization methods will be discussed in more detail below.

##### Y-standardization

*Y*-standardization replaces the original scale of the dependent variable with standard deviations (SDs) [15]. After *y*-standardization, the dependent variable has a standard deviation of 1. When *y*-standardization is applied to the coefficients from multiple logistic regression models with the same dependent variable, the variance of this dependent variable will become comparable across the models. After *y*-standardization, the coefficients represent the SDs change in the dependent variable for a one unit change in the independent variable. To perform *y*-standardization, the coefficients from eqs. 1, 2, and 3 are divided by the SD of the dependent variable in that equation. The SDs of the dependent variables in eqs. 1, 2, and 3 can be derived using eqs. 4, 5, and 6, respectively [2, 6].

4 $$ SD(Y1)=\sqrt{c^2 VAR(X)+{\pi}^2/3} $$

5 $$ SD(M2)=\sqrt{a^2 VAR(X)+{\pi}^2/3} $$

6 $$ SD(Y3)=\sqrt{{c^{\prime}}^2VAR(X)+{b}^2VAR(M)+2b{c}^{\prime }COV(XM)+{\pi}^2/3} $$

Where in Eq. 4, *SD*(*Y*1) represents the SD of the outcome in eq. 1, *c* represents the *c* coefficient in eq. 1, and *VAR*(*X*) represents the variance of the exposure. In eq. 5, *SD*(*M*2) represents the SD of the mediator in eq. 2, *a* represents the *a* coefficient in equation 2, and *VAR*(*X*) represents the variance of the exposure. In eq. 6, *SD*(*Y*3) represents the SD of the outcome in eq. 3, *c*^′^ represents the *c’* coefficient from equation 3, *VAR*(*X*) is the variance of the exposure, *b* represents the *b* coefficient from equation 3, *VAR*(*M*) represents the variance of the mediator, and *COV*(*XM*) represents the covariance between the exposure and mediator. In all equations *π* equals the number pi.

##### Full-standardization

Full-standardization replaces both the scale of the dependent and independent variable with SDs [15]. Therefore, the SD of both the independent and dependent variable will be 1. After full-standardization, the coefficients represent the SDs change in the dependent variable for one SD increase in the independent variable. However, it is important to note that this interpretation does not make sense when the exposure is dichotomous, for example one SD change in a treatment [15]. To perform full-standardization, the coefficients from eqs. 1, 2, and 3 are multiplied by the SD of the independent variable and then divided by the SD of the dependent variable. The SDs of the independent variables can be derived in the ordinary way, and the SDs of the dependent variables can be derived using eqs. 4, 5, and 6.

##### The standardized logistic solution

The standardized logistic solution replaces the scale of the *c* coefficient with the scale of the *c’* coefficient using eq 7. [1, 2].

7 $$ {c}_{standardized}=c\sqrt{1+\frac{b^2{\sigma}_{MX}^2}{\pi^2/3}} $$

Where *c*_*standardized*_ is the standardized *c* coefficient, *c* is the *c* coefficient from eq. 1, *b* is the *b* coefficient from eq. 3, ${\sigma}_{MX}^2$ is the residual variance from eq. 2, and *π*^2^/3 is the error variance of the standard logistic distribution with *π* representing the number pi. Because in logistic regression no residual variance is being estimated, the standardized logistic solution can only be applied when the mediator is continuous.

#### Potential outcomes framework

The potential outcomes framework provides definitions of the mediated effect that can be used to decompose the total effect of an exposure on an outcome into causal direct and indirect effects [5]. The potential outcomes framework therefore explicitly assumes that there are no unobserved confounders of the relationships in the mediation model. There are several ways in which the potential outcomes framework can be used to estimate direct and indirect effects [16–18]. In this paper we focus on the logistic-regression based method as described by VanderWeele and Vansteelandt [18]. Under the assumption of no unobserved confounders, no exposure-mediator interaction, and a low outcome prevalence (i.e. 10% or lower), the indirect effect for mediation models with a dichotomous outcome is defined as the product of the *a* and *b* coefficients from eqs. 2 and 3 [19]. Furthermore, in this situation, the direct effect equals the *c’* coefficient from eq. 3. Under the no unobserved confounders and no exposure-mediator interaction assumptions, the indirect and direct effect odds ratios for mediation models with a dichotomous mediator and outcome can be calculated using eqs. 8 and 9 [19].

8 $$ Indirect\ effect\ OR=\frac{\left(1+\exp \left({i}_2\right)\right)\left(1+\exp \left(b+{i}_2+a\right)\right)}{\left(1+\exp \left({i}_2+a\right)\right)\left(1+\exp \left(b+{i}_2\right)\right)} $$

9 $$ Direct\ effect\ OR=\exp \left({c}^{\prime}\right) $$

Where *i*_2_ represents the intercept from eq. 2, *b* represents the *b* coefficient from eq. 3, *a* represents the *a* coefficient in eq. 2, and *c*^′^ represents the *c’* coefficient from eq. 3.

The total effect is defined as either the product of the direct and indirect effect when the effect estimates are on the odds ratio scale, or as the summation of the direct and indirect effect when the effect estimates are on the log odds ratio scale [19].

## Results

### Simulation study

Tables 2, 3, and 4 show the results of the simulation study comparing the performance of multiple regression, SEM, and the potential outcomes framework. Since the estimates yielded by multiple regression and SEM were exactly the same across all conditions, the results of these two methods are presented together.

**Table 2:** Bias and efficiency yielded by the three compared methods for models with a continuous mediator

		Multiple regression and SEM								Potential outcomes	
		Crude		*y*-standardization		Full-standardization		Standardized logistic solution			
*Y prev*		*bias*	*MSE*	*bias*	*MSE*	*bias*	*MSE*	*bias*	*MSE*	*bias*	*MSE*
0.5	Indirect effect									−0.001	0.002
	*ab*	−0.001	0.002	−0.001	0.000	−0.000	0.001	−0.001	0.002		
	*c-c’*	−0.068	0.006	−0.008	0.000	−0.036	0.002	−0.021	0.003		
	Proportion mediated									0.003	0.003
	*ab*/(*ab* + *c’*)	0.003	0.003	0.003	0.003	0.065	0.009	0.003	0.003		
	*ab*/*c*	0.032	0.005	0.009	0.003	0.073	0.010	0.011	0.003		
	1-(*c’*/*c*)	−0.044	0.004	− 0.008	0.003	−0.008	0.003	−0.010	0.003		
0.3	Indirect effect									0.000	0.003
	*ab*	0.000	0.003	−0.000	0.000	−0.000	0.001	0.000	0.003		
	*c-c’*	−0.061	0.005	−0.005	0.001	−0.033	0.002	−0.012	0.003		
	Proportion mediated									0.001	0.004
	*ab*/(*ab* + *c’*)	0.001	0.004	0.001	0.004	0.064	0.010	0.001	0.004		
	*ab*/*c*	0.027	0.005	0.005	0.004	0.069	0.010	0.006	0.004		
	1-(*c’*/*c*)	−0.041	0.005	−0.006	0.004	−0.006	0.004	−0.007	0.004		
0.1	Indirect effect									0.003	0.006
	*ab*	0.003	0.006	0.001	0.001	0.001	0.001	0.003	0.006		
	*c-c’*	−0.036	0.005	0.005	0.001	−0.023	0.002	0.016	0.003		
	Proportion mediated									0.006	0.008
	*ab*/(*ab* + *c’*)	0.006	0.008	0.006	0.008	0.070	0.017	0.006	0.008		
	*ab*/*c*	0.023	0.010	0.002	0.008	0.065	0.015	0.001	0.008		
	1-(*c’*/*c*)	−0.020	0.008	0.011	0.009	0.011	0.009	0.013	0.009		

*Abbreviations*: *SEM* structural equation modeling, *Y prev* outcome prevalence, *MSE* mean squared error

**Table 3:** Bias and efficiency yielded by the three compared methods for models with a dichotomous mediator

			Multiple regression and SEM						Potential outcomes	
			Crude		*y*-standardization		Full-standardization			
*M prev*	*Y prev*		*bias*	*MSE*	*bias*	*MSE*	*bias*	*MSE*	*bias*	*MSE*
0.5	0.5	Indirect effect							0.000	0.000
		*ab*	0.002	0.008	0.000	0.001	0.000	0.000		
		*c-c’*	−0.287	0.083	−0.054	0.003	0.006	0.000		
		Proportion mediated							0.001	0.001
		*ab*/(*ab* + *c’*)	−0.003	0.005	−0.136	0.021	−0.235	0.057		
		*ab*/*c*	0.167	0.048	−0.096	0.014	−0.236	0.057		
		1-(*c’*/*c*)	−0.266	0.072	−0.254	0.066	−0.254	0.066		
	0.3	Indirect effect							−0.001	0.000
		*ab*	0.003	0.010	−0.001	0.001	0.000	0.000		
		*c-c’*	−0.288	0.083	−0.055	0.003	0.007	0.000		
		Proportion mediated							0.001	0.001
		*ab*/(*ab* + *c’*)	−0.004	0.006	−0.136	0.022	−0.235	0.057		
		*ab*/*c*	0.168	0.054	−0.095	0.016	−0.235	0.057		
		1-(*c’*/*c*)	−0.265	0.071	−0.254	0.066	−0.254	0.066		
	0.1	Indirect effect							0.002	0.001
		*ab*	0.016	0.021	−0.003	0.001	0.002	0.000		
		*c-c’*	−0.284	0.081	−0.052	0.003	−0.004	0.000		
		Proportion mediated							0.004	0.002
		*ab*/(*ab* + *c’*)	−0.001	0.012	−0.132	0.024	−0.230	0.056		
		*ab*/*c*	0.187	0.087	−0.087	0.021	−0.231	0.057		
		1-(*c’*/*c*)	−0.261	0.070	−0.248	0.064	−0.248	0.064		
0.3	0.5	Indirect effect							−0.001	0.001
		*ab*	−0.001	0.020	−0.001	0.001	−0.000	0.000		
		*c-c’*	−0.299	0.081	−0.062	0.003	− 0.009	0.000		
		Proportion mediated							−0.001	0.001
		*ab*/(*ab* + *c’*)	−0.006	0.013	−0.138	0.025	−0.247	0.062		
		*ab*/*c*	0.171	0.086	−0.093	0.022	−0.246	0.062		
		1-(*c’*/*c*)	−0.283	0.070	−0.272	0.066	−0.272	0.066		
	0.3	Indirect effect							−0.001	0.001
		*ab*	−0.001	0.011	−0.001	0.001	0.000	0.000		
		*c-c’*	−0.294	0.087	−0.060	0.004	−0.007	0.000		
		Proportion mediated							0.001	0.001
		*ab*/(*ab* + *c’*)	−0.003	0.007	−0.135	0.022	−0.244	0.061		
		*ab*/*c*	0.176	0.061	−0.090	0.016	−0.244	0.061		
		1-(*c’*/*c*)	−0.274	0.076	− 0.264	0.071	−0.264	0.071		
	0.1	Indirect effect							0.003	0.001
		*ab*	0.012	0.010	0.002	0.001	0.001	0.000		
		*c-c’*	−0.283	0.090	−0.054	0.004	−0.001	0.000		
		Proportion mediated							0.004	0.003
		*ab*/(*ab* + *c’*)	−0.004	0.006	− 0.134	0.022	−0.242	0.062		
		*ab*/*c*	0.179	0.054	−0.090	0.015	−0.244	0.062		
		1-(*c’*/*c*)	−0.260	0.081	−0.252	0.075	−0.252	0.075		
0.1	0.5	Indirect effect							0.004	0.001
		*ab*	0.013	0.026	0.002	0.002	−0.018	0.000		
		*c-c’*	−0.333	0.111	−0.082	0.007	−0.032	0.001		
		Proportion mediated							0.004	0.001
		*ab*/(*ab* + *c’*)	−0.006	0.011	−0.137	0.025	−0.284	0.082		
		*ab*/*c*	0.223	0.113	−0.066	0.020	−0.281	0.081		
		1-(*c’*/*c*)	−0.332	0.111	−0.326	0.107	−0.326	0.107		
	0.3	Indirect effect							0.003	0.001
		*ab*	0.009	0.025	0.002	0.002	−0.018	0.000		
		*c-c’*	−0.328	0.108	−0.080	0.006	−0.030	0.001		
		Proportion mediated							0.005	0.001
		*ab*/(*ab* + *c’*)	−0.005	0.013	−0.135	0.025	−0.283	0.082		
		*ab*/*c*	0.225	0.121	−0.065	0.022	−0.281	0.081		
		1-(*c’*/*c*)	−0.323	0.105	−0.318	0.102	−0.318	0.102		
	0.1	Indirect effect							0.003	0.001
		*ab*	0.002	0.033	−0.001	0.002	−0.018	0.001		
		*c-c’*	−0.319	0.102	−0.076	0.006	−0.026	0.001		
		Proportion mediated							0.003	0.002
		*ab*/(*ab* + *c’*)	−0.024	0.027	−0.145	0.033	−0.286	0.084		
		*ab*/*c*	0.193	0.130	−0.081	0.031	−0.285	0.084		
		1-(*c’*/*c*)	−0.310	0.098	−0.307	0.096	−0.307	0.096		

*Abbreviations*: *SEM* structural equation modeling, *M prev* mediator prevalence, *Y prev* outcome prevalence, *MSE* mean squared error

**Table 4:** Application of the three compared methods to the real-life data examples

		Multiple regression and SEM				Potential outcomes^a^
		Crude	*y*-standardization	Full-standardization	Standardized logistic solution^b^	
Situation 1 M continuous Y dichotomous	Total effect (*c*)	−0.20	−0.11	−0.20	−0.21	−0.21
	*a* coefficient^d^	0.67	0.67	0.67	0.67	0.67
	*b* coefficient	−0.04	−0.02	−0.23	−0.04	−0.04
	Direct effect (*c’*)	−0.19	−0.10	−0.18	−0.19	−0.19
	Indirect effect					−0.03
	*ab*	−0.03	−0.01	−0.15	−0.03	
	*c-c’*	−0.02	−0.01	−0.02	−0.02	
	Proportion mediated					0.12
	*ab*/(*ab* + *c’*)	0.12	0.12	0.47	0.12	
	*ab*/*c*	0.13	0.12	0.77	0.12	
	1-(*c’*/*c*)	0.09	0.11	0.11	0.11	
Situation 2 M dichotomous Y dichotomous	Total effect (*c*)	−0.20	−0.11	−0.20	NA	−0.21
	*a* coefficient	0.11	0.06	0.11	NA	0.11
	*b* coefficient	−0.60	−0.32	−0.16	NA	−0.60
	Direct effect (*c’*)	−0.19	−0.10	−0.19	NA	−0.19
	Indirect effect					−0.01
	*ab*	−0.06	−0.02	−0.02	NA	
	*c-c’*	−0.01	−0.01	−0.01	NA	
	Proportion mediated					0.07
	*ab*/(*ab* + *c’*)	0.25	0.16	0.09	NA	
	*ab*/*c*	0.32	0.17	0.09	NA	
	1-(*c’*/*c*)	0.06	0.07	0.07	NA	

*Abbreviations: SEM* structural equation modeling, *M* mediator variable, *Y* outcome variable, *NA* not available

^a^The output of the potential outcomes framework contains odds ratios, the coefficients in the table are log transformed to make the coefficients comparable to the coefficients yielded by multiple regression and SEM

^b^The standardized logistic solution cannot be applied to mediation models with a dichotomous mediator variable

^d^The *a* coefficient is based on linear regression

#### Continuous mediator

When the mediator was continuous (Table 2), the estimates based on the potential outcomes framework and the crude indirect effect *ab* and proportion mediated *ab/(ab + c’)* based on multiple regression and SEM generally had the lowest bias and highest efficiency. All standardization methods decreased bias and increased efficiency of the *c*-*c’* estimate compared to the crude *c-c’* estimate based on multiple regression and SEM. Y-standardization and the standardized logistic solution both decreased bias and increased efficiency of the *ab/c* and 1-(*c’/c*) estimates compared to the crude *ab/c* and 1-(*c’/c*) estimates based on multiple regression and SEM. However, full-standardization was not able to decrease bias and increase efficiency in the proportion mediated estimates based on multiple regression and SEM. These results were observed across all three outcome prevalences.

#### Dichotomous mediator

When the mediator is dichotomous (Table 3), the estimates based on the potential outcomes framework and the crude indirect effect *ab* and proportion mediated *ab/(ab + c’)* based on multiple regression and SEM are both unbiased with respect to their own true values. The standardization methods did decrease bias and increase efficiency in the *c-c’* estimate, but the performance of the standardized proportion mediated estimates was worse than the performance of the crude proportion mediated estimates based on multiple regression and SEM. Even though the estimates based on the potential outcomes framework and the crude indirect effect *ab* and proportion mediated *ab/(ab + c’)* based on multiple regression and SEM are unbiased and efficient with respect to their own true values, differences were observed between the effect estimates based on the potential outcomes framework and multiple regression and SEM.

### Real-life data examples

Table 4 shows the results yielded for the real-life data examples. As in the simulation study, multiple regression and SEM yielded exactly the same results. When the mediator was continuous, the estimates of the indirect effect (−0.03) and proportion mediated (0.12) in the potential outcomes framework equaled the crude indirect effect *ab* and the proportion mediated *ab*/(*ab* + *c’*) in multiple regression and SEM. The indirect effect of −0.03 corresponds to an odds ratio of 0.97, which indicates that for one unit increase in the sum of four skinfolds the odds of being in the high CD group decreases by a factor of 0.97 via an increase in average heart rate. This indirect effect explained 12% of the total effect of sum of four skinfolds on CD.

When the mediator was dichotomous, the crude *ab* and *ab*/(*ab* + *c’*) estimates based on multiple regression and SEM were −0.06 and 0.25 respectively. This indirect effect estimate corresponds to an odds ratio of 0.94, indicating that for one unit increase in the sum of four skinfolds the odds of being in the high CD group decreases by a factor of 0.94 via an increased odds of being in the high average heart rate group. This indirect effect explained 25% of the total effect of sum of four skinfolds on CD. The indirect effect and proportion mediated based on the potential outcomes framework were − 0.01 and 0.07 respectively. This indirect effect estimate corresponds to an odds ratio of 0.99, indicating that for one unit increase in the sum of four skinfolds the odds of being in the high CD group decreases by a factor of 0.99 via an increased odds of being in the high average heart rate group. This indirect effect explained 7% of the total effect of sum of four skinfolds on CD.

## Discussion

The aim of this paper was to show the relative performance of different methods to estimate the indirect effect and proportion mediated for mediation models with a dichotomous outcome. The effect estimates based on the potential outcomes framework and the crude indirect effect estimate *ab* and the crude proportion mediated *ab*/(*ab* + *c’*) based on multiple regression and SEM perform well in all situations. When the mediator was continuous, the effect estimates in the potential outcomes framework and in multiple regression/SEM coincided, but this was not the case when the mediator was dichotomous. Standardization of the coefficients from multiple regression/SEM prior to estimating the indirect effect and the proportion mediated does generally not increase the performance of these estimates.

For both models with a continuous or a dichotomous mediator and across all prevalence rates of the mediator and outcome, the crude indirect effect estimate *c*-*c’* and the crude estimates of the proportion mediated *ab*/*c* and 1-(*c’*/*c*) performed worse than the crude *ab* and *ab*/(*ab* + *c’*) estimates. We found that, compared to the crude estimates, standardization only decreased bias and increased efficiency in the *c*-*c’* estimate of the indirect effect and the *ab*/*c* and 1-(*c’*/*c*) estimates of the proportion mediated. In line with our findings, previous studies only advised standardization of the coefficients when calculating the indirect effect as *c*-*c*’ and the proportion mediated as *ab*/*c* or 1-(*c’*/*c*) [2–4]. This is advice is relevant when the indirect effect is phrased in terms of a difference in coefficients [2]. Furthermore, when the mediator was dichotomous, the standardized estimates of the proportion mediated performed worse than the crude estimates in terms of bias and efficiency. Furthermore, it is important to note that both *y*-standardization and full-standardization may hamper a clinically meaningful interpretation of the indirect effect [15].

That multiple regression and SEM yielded exactly the same estimates of the indirect effect and proportion mediated can be explained by their mathematical equivalence [20]. Furthermore, when the mediator is continuous and in the absence of exposure-mediator interaction, the formulas for calculating the indirect effect and proportion mediated in the potential outcomes framework are mathematically equivalent to the *ab* and *ab*/(*ab* + *c’*) estimates in multiple regression and SEM [18]. However, when the mediator is dichotomous, there is a discrepancy between the indirect effect estimate in the potential outcomes framework and in multiple regression and SEM. This discrepancy is caused by the differences in the formulas of the indirect effect used by the two methods when the mediator is dichotomous [21]. Further research is needed to assess why and when these two formulas lead to different indirect effect estimates.

### Change of scales in logistic models

The systematic underestimation of the *c*-*c’* estimate of the indirect effect can be explained by the change of scales of the coefficients in nested logistic models. The scale of the coefficients in logistic models is dependent on the total variance of the dependent variable [3]. The total variance in a variable is a combination of explained and unexplained variance. When a particular variable is added to a linear regression model, the unexplained variance decreases with the same amount as the explained variance increases. However, in a logistic regression model a standard logistic distribution is assumed, in which the unexplained variance is fixed at 3.29 [22]. So, the total amount of variance in the dependent variable must increase when an added variable explains some of the variance in the dependent variable. Consequently, also the scale of the coefficients in the model will increase.

The change of scales becomes a problem when mediation is investigated. Suppose we add a potential mediator, that is highly related to the outcome, to a logistic regression model with an exposure variable. The strong relationship between the mediator and outcome variable will force the total amount of variance in the outcome variable to increase. To deal with this increased total variance, the scale of the coefficients in the model will increase as well. This increase in the coefficient for the exposure variable would also happen when there is no mediation at all, i.e. when the relationship between the exposure and mediator variable is equal to zero. In that case the increase in the coefficient for the exposure variable would be completely attributable to the increase in the total amount of variance in the outcome variable and not to mediation [23].

When there truly is mediation, the change of scales in logistic models will bias the *c*-*c’* estimate of the indirect effect. Because the mediator explains at least a part of the total effect of the exposure on the outcome, the direct effect (*c’* coefficient) is expected to be lower than the total effect (*c* coefficient). However, at the same time the magnitude of the coefficient for the direct effect will increase because of the addition of the mediator to the model. Consequently, the *c*-*c’* estimate will be a systematic underestimation of the true (positive) indirect effect. Previous simulation studies showed that the magnitude of this underestimation depends on both the strength of the relationship between the mediator and outcome, and on the sample size [2, 3]. Furthermore, it is important to note that even when the true mediated effect equals zero, the indirect effect based on *c-c’* will likely be nonzero and thus a misleading estimate of the true indirect effect.

### Significance testing

Often researchers are interested in using statistical tests to test for the presence of a mediated effect. Furthermore, it has been suggested that when the outcome prevalence is higher than 10%, the indirect effect estimates can only be used to test for the presence of a mediated effect instead of interpreting the indirect effect estimate itself [21]. It should, however, be noted that the statistical significance of an indirect effect does not say anything about its clinical relevance [24]. The clinical relevance of an indirect effect can only be assessed through its magnitude. Unfortunately, the magnitude of the indirect effect based on logistic models will often be affected by unobserved heterogeneity. To avoid the problem of unobserved heterogeneity in the interpretation of the indirect effect, the use of alternative models has been proposed, such as linear probability models, average marginal effects models, and log-linear models [19, 22]. Further research is needed to assess the usefulness of these models for mediation analysis with a dichotomous outcome.

### Strengths and limitations

To our knowledge this is the first paper extensively comparing unstandardized and standardized estimates of the indirect effect and proportion mediated based on multiple regression, SEM, and the potential outcomes framework for models with a dichotomous outcome. In our simulation study we assessed multiple conditions based on the prevalence of the mediator and outcome, as the potential outcomes framework assumes the outcome to be rare. Our study showed that the bias and efficiency of the estimates of the indirect effect and proportion mediated across all prevalence rates are low. However, it is important to note that the odds ratios from the potential outcomes framework won’t approximate risk ratios for high prevalence rates, i.e. 10% to 50% [18].

For the sake of simplicity, we did not include confounders in the simulated models. However, we believe that the results in this paper also apply for models that do include confounders. In practice it is important to consider potential confounders of all relationships in the mediation model. In all three methods compared in this paper, the estimates of the indirect effect and proportion mediated can be adjusted for confounding by adding the potential confounders to all fitted regression equations [25–27].

## Conclusions

In general, standardization of the coefficients prior to estimating the indirect effect and the proportion mediated may not increase the performance of these estimates. We therefore recommend to either use the estimates based on the potential outcomes framework or the crude *ab* estimate and *ab*/(*ab* + *c’*) estimate of the indirect effect and proportion mediated, respectively, based on multiple regression and SEM. For models with a continuous mediator, these estimates from multiple regression and SEM coincide with the estimates from the potential outcomes framework. When the mediator is dichotomous, the estimates based on the potential outcomes framework deviate from the estimates based on multiple regression and SEM. Further research is needed to assess why and when these methods lead to different effect estimates.

## Abbreviations

AB: Absolute bias
CD: Carotid distensibility
MSE: Mean squared error
NA: Not available
PREV: Prevalence
SD: Standard deviation
SEM: Structural equation modeling
VAR: Variance
